# Infants Generalize Beliefs Across Individuals

**DOI:** 10.3389/fpsyg.2020.547680

**Published:** 2020-09-22

**Authors:** Kimberly Burnside, Cassandra Neumann, Diane Poulin-Dubois

**Affiliations:** Department of Psychology, Concordia University, Montréal, QC, Canada

**Keywords:** theory of mind, infancy, violation-of-expectation, false belief, true belief

## Abstract

It has been argued that infants possess a rich, sophisticated theory of mind (ToM) that is only revealed with tasks based on spontaneous responses. A mature (ToM) implies the understanding that mental states are person specific. Previous studies on infants’ understanding of motivational mental states, such as goals and preferences have revealed that, by 9 months of age, infants do not generalize these motivational mental states across agents. However, it remains to be determined if infants also perceive epistemic states as person specific. Therefore, the goal of the present study was to use a switch agent paradigm with the classic false belief violation-of-expectation task. Results revealed that 16-month-old infants attributed true and false beliefs to a naïve agent – they did not perceive beliefs as person specific. These findings indicate that the mechanisms that underlie infants’ implicit attribution of beliefs differ from those assumed for explicit reasoning about beliefs.

## Introduction

The depth of infants’ theory of mind (ToM) is currently the subject of a heated debate. For decades, researchers have attempted to determine exactly when this foundational socio-cognitive ability develops. Traditionally, ToM was thought to emerge between 3 and 5 years of age (Wellman et al., 2001; Wellman and Liu, 2004). Over the past decades, a large number of studies have challenged this view by providing evidence for early ToM understanding in infancy using tasks that have minimal processing demands (Clements and Perner, 1994; Gergely et al., 1995; Onishi and Baillargeon, 2005; Phillips and Wellman, 2005; Scott, 2017). These implicit tasks, which measure infants’ spontaneous looking or actions, provided further insight into precocious ToM in infants as young as 7 months of age. However, the interpretation of findings based on these implicit ToM tasks is currently the focus of an intense debate. One side of the debate (i.e., the rich and mentalistic view), founded on the numerous studies that demonstrate an early understanding of ToM, argues that infants have an adult-like understanding of ToM and that this ability can be reliably measured using implicit tasks (Baillargeon et al., 2010, 2016, 2018; Scott and Baillargeon, 2017). Conversely, other researchers support leaner interpretations of infants’ behaviors measured by these tasks (Apperly and Butterfill, 2009; Ruffman, 2014; Heyes, 2014a; Poulin-Dubois et al., 2018). Given the current relevance of this debate, the goal of the current study is to determine if the construct measured by implicit tasks corresponds to a fully formed, sophisticated ToM understanding – equivalent to the ToM understanding found in preschoolers and adults using explicit, elicited-response tasks as is suggested by the mentalistic view.

ToM is defined as the understanding that oneself and others have mental states that guide our behaviors (Wellman, 2014). As such, it is supposed to play an essential role in human interactions – it is an ability that permits us to understand another person’s perspective and to behave accordingly. ToM is an umbrella term that covers several different sub-concepts, such as desires, intentions, and beliefs, that permit us to understand others’ mental states (Wellman and Liu, 2004). A fully formed understanding of beliefs requires mastery of true and false belief scenarios. A true belief is when an individual’s belief is congruent with reality. A false belief is when an individual’s belief is incongruent with reality (e.g., believing an object is in a location when in fact it is in another location). A classic false belief task is the Sally-Anne task, which measures whether children can answer where Sally will look for her marble after Anne changed the marble’s location without her knowing (Baron-Cohen et al., 1985). This explicit task requires a verbal response on the part of the participant. In contrast, implicit false belief tasks rely on the participant’s spontaneous looking behavior (e.g., time spent looking at the scene). In a landmark study, Onishi and Baillargeon (2005) used a violation-of-expectation (VOE) task to test belief understanding in 15-month-olds. VOE tasks assess whether infants look longer (i.e., find it surprising) when an agent acts in a way that is inconsistent with her beliefs. During a series of familiarization trials, infants saw an agent play with a toy and then place it inside a green box. In the belief-induction trial, the toy changed location (e.g., to a yellow box) with either the agent present, inducing a true belief, or absent, changing the agent’s belief to a false belief. During the test trials, half of the infants saw the agent reach into the green box and the other half into the yellow box. If the infants expected the agent to search for her toy on the basis of her belief about its location (and not its actual location), then the infants should look longer when that expectation was violated. Results indicated that infants’ looking times were coherent with the hypothesis that they were attributing both true and false beliefs. Baillargeon et al. (2010, p. 110) concluded that “false-belief understanding provides evidence for a sophisticated (and possibly uniquely human) ability to consider the information available to an agent when interpreting and predicting the agent’s actions – even if this information is inaccurate and incompatible with one’s own.” According to this rich view, infants and young children fail the traditional explicit ToM tasks because these tasks are heavily based on language abilities and executive functions, rather than due to an undeveloped ToM (Baillargeon et al., 2010; Setoh et al., 2016; Scott, 2017). This “processing-demands” account argues that, in explicit tasks, children must first select the correct response (response-selection process), inhibit the response of the actual location of the object (response-inhibition process), as well as remembering the agent’s false belief (working memory; but see Rubio-Fernández et al., 2017 and Fenici and Garofoli, 2020 for arguments challenging this view). Others have suggested that a verbal false-belief task involves a complex interplay between executive decision-making, the language faculty, and mind reading (Carruthers, 2013), whereas others argue that such task requires well-developed pragmatic skills because there will generally be three interpretations of the question that are activated, competing to control the answer. One is that the child is being invited to be helpful toward the protagonist. Another is that she is being asked to exhibit her knowledge of the events that have unfolded in the story. The third is that she is supposed to exhibit her knowledge of the way in which the protagonist’s beliefs will issue in action (Westra and Carruthers, 2017; but see Fenici, 2016 for an in-depth discussion of how social experiences induce success in traditional false belief tasks).

There are several proposals that posit leaner interpretations of behaviors observed with implicit ToM paradigms (see Krupenye and Call, 2019 for a brief summary). For example, Apperly and Butterfill (2009) argue that infants’ behaviors observed in implicit tasks might not be based on the same mechanisms as those in older children and adults, but rather reflect a separate ToM system altogether that develops independently (i.e., Minimalist account). Specifically, it has been proposed that there is an “efficient mindreading system [that] is evolutionarily and ontogenetically ancient, operates quickly, and is largely automatic and independent of central cognitive resources” (i.e., System 1), and a “flexible mindreading system [that] develops late, operates slowly, and makes substantial demands on executive control processes” (i.e., System 2; Low et al., 2016). If this is the case, then infants’ looking behaviors in implicit tasks might measure a more primitive ability than what is believed to be a fully formed ToM understanding. On the other hand, researchers like Ruffman (2014) believe that infants’ responses in VOE tasks can be explained by the learning of simple behavioral rules (i.e., Behavioral Rule account) whereby infants rely on rules such as “people look for an object where they last saw it and not necessarily where the object actually is” (Perner and Ruffman, 2005, p. 215). This rule is known and applied based on perceived behavior without any inference about mental states. Another lean view proposes that infants are solving implicit false belief tasks by submentalizing (Heyes, 2014b). Submentalizing is when individuals’ behaviors seem as though they are thinking about mental states but instead, their looking patterns are a result of the violation of the participants’ expectations of superficial associations created in the previous trials (i.e., perceptual novelty of the test trial). In other words, infants are simply responding to the novelty of the configuration of colors, shapes, and movements and are not attributing mental states to the agent.

The nature of the mechanisms involved in infants’ reasoning during the VOE task was recently examined in infants and adults in a conceptual replication – a live human agent was replaced by a live inanimate agent (i.e., a toy crane lacking all morphological animacy; Burnside et al., 2019). Results of Onishi and Baillargeon (2005) with a human agent were replicated, suggesting that 16-month-old infants generalize the attribution of false beliefs to an inanimate agent that displays agentive properties. Adults, however, did not attribute a belief to the toy crane. Similar results were observed by Tauzin and Gergely (2018) who found that infants generalized mental states to blobs. Although a plausible explanation for this replication is that infants respond to the novelty of the perceptual features of the scene seen in the test trial regardless of the agent (i.e., submentalizing), another plausible interpretation is that infants attribute mental states to all objects that they identify as agents. It has been argued that the mentalistic view does not posit that early mental-state reasoning is restricted to *animate objects*, but to *agents*, which are objects that display cues such as autonomous motion, goal-oriented, contingent, and action-at-a-distance behaviors (Carey, 2000; Scholl and Tremoulet, 2000; Surian and Franchin, 2020). Furthermore, proponents of the rich view argue that many of the lean views outlined earlier can be ruled out by arguing that mental state attribution is a more parsimonious interpretation of findings across studies than multiple rules for each false belief task or for a lack of evidence that low-level variables, such as the reappearance of the agent in the change of location false belief task, is disruptive (see Scott and Baillargeon, 2014 Baillargeon et al., 2018 for arguments against lean views). However, it has been argued that the criterion of parsimony does not automatically endorse mentalist over behavior-reading accounts. Parsimony can ask us to reduce either the number of rules or the number of concepts necessary to explain infants’ performance in implicit false belief tests. Furthermore, parsimony will privilege mind‐ or behavior-reading accounts depending on how rules are conceptualized. Some level of rule abstraction is within the grasp on infants in the categorization of both the environmental stimuli and the agent’s reactions (Low and Wang, 2011; Ruffman, 2014; Fenici, 2015). Needless to say, the lack of consensus has fueled a heated debate.

If, in fact, infants are overattributing mental states, then this would suggest that the psychological mechanisms that underlie infants’ behaviors in implicit tasks such as the VOE might differ from those at play when older children and adults attribute beliefs. Given that a mature ToM involves understanding that mental states are person specific (Wellman, 1990), another way to assess the maturity of infants’ ToM is to examine if infants generalize beliefs from a knowledgeable to an ignorant agent (i.e., an agent who has not witnessed an event). If infants generalize such beliefs, then their understanding of beliefs is not as mature as that of older children and adults since both groups understand that thoughts are not transferred across individuals without some form of communication.

This hypothesis has been tested in the case of simple, motivational mental states. Buresh and Woodward (2007) used a switch agent version of the visual habituation paradigm to test 13-month-olds’ ability to track goals. First, they familiarized infants with two actors who looked noticeably different. In the habituation trials, infants were shown an actor repeatedly playing with an object until the infant habituated to the scene. At test, infants in the single-actor condition looked longer, as expected, in the new-goal trials (i.e., actor reached for a different object) than in the new-side trials (i.e., actor reaches for the same object as in the habituation trials). The infants in the switch-actor condition looked equally long during the new-goal and new-side trials. This suggests that infants were able to understand that a goal belongs to a particular person and that this goal cannot be transferred to others (i.e., person specific). Henderson and Woodward (2012) conducted a similar paradigm – they used a habituation paradigm with 9-month-old infants. In the training phase, infants viewed an event during which an experimenter demonstrated a clear preference for one of two novel objects. Then, the infants were administered a habituation phase during which the experimenter repeatedly referred to his or her preferred object. Finally, the infants were administered a phase during which the initial experimenter (Same Actor condition) or the new experimenter (Switch Actor condition) alternately picked the target object and the distractor object six times while consistently labeling the objects. The authors found that infants did not generalize object preference to the new experimenter, suggesting that 9-month-old infants understand that preferences are person-specific.

Kampis et al. (2013) used a similar switch agent procedure to assess 10-month-old’s understanding of preference – referred to as “attitude”. In this paradigm, infants were either assigned to an “occlusion” group or to a “no-occlusion” group. First, infants were shown an agent (Agent A) and two objects placed in front of her, but behind translucent barriers. Infants in the no-occlusion group saw a hand remove one of the two objects. After this, Agent A reached for the remaining object – because this object was the only available object for Agent A to take, no preference could be inferred. However, infants in the occlusion group saw a hand place an opaque barrier between the object and Agent A. Then, the hand removed the object located behind this barrier – Agent A did not see the removal of this object and “thinks” that both objects are available. Following this, Agent A reached for the object that was not occluded – as such, it is assumed that this object is preferred because it is inferred that Agent A made a choice. At test, both groups saw a different agent (i.e., Agent B) reach for one of the two objects – consistent or inconsistent with Agent A’s choice. Only the infants in the occlusion group looked longer when Agent B’s choice was inconsistent with Agent A’s choice. This implies that they were surprised that Agent B chose a different object from Agent A, suggesting that infants generalized Agent A’s preference to Agent B. Therefore, Kampis et al. (2013) provided some evidence showing that infants do not make person-specific preference attributions.

The studies using the switch-agent paradigm all assessed infants’ understanding of motivational states, such as goals and preferences. However, to the best of our knowledge, none has examined if infants also treat *epistemic* states as person-specific. In other words, it is still unclear how infants would behave to a change of agent in a typical false belief paradigm. Given the ongoing debate about the mechanisms of false belief attribution in infancy, it is crucial to investigate whether infants understand that beliefs are unique to individuals – a marker of a mature, adult-like ToM understanding. Therefore, the main goal of the present study is to determine whether infants generalize *beliefs* across individuals in the classic VOE false belief task.

Specifically, infants watched an agent interact with an object followed by a belief-induction trial that induced a true or false belief to this agent. At test, a naïve agent, never exposed to the location of the toy, reached in one of the two boxes. If there is a crucial limitation in the mechanisms that infants tacitly use to reason about other agents’ actions and, possibly, their minds, they should form the expectation that the naïve agent possesses a belief, failing to recognize that beliefs require perceptual access to the object during the familiarization trials – and that such experience is not transferable across different individuals. Conversely, if infants have no expectation about the naïve agent’s actions in the test trial, then those in the congruent group should look equally long as those in the incongruent group (here, the terms congruent and incongruent are based on the initial agent’s beliefs to keep consistent with the original study). Therefore, if infants reason about beliefs with the mechanisms attributed to older children, then we expect no group differences in looking time. However, if infants’ looking patterns replicate those found in Onishi and Baillargeon’s (2005) study (i.e., longer looking in the incongruent group compared with the congruent group), then infants generalize beliefs from knowledgeable to ignorant agents. Finally, as a manipulation check, a filmed version of this paradigm was shown to preschoolers and to adult participants to confirm whether they would attribute ignorance to the naïve agent and predict a random search behavior.

## Materials and Methods

### Participants

An *a priori* power analysis required 48 infant participants per belief condition to obtain a moderate-strong effect size (*d* = 0.90) and adequate power (1 − *β* = 0.85). This target effect size was taken from previous research (Burnside et al., 2019).

#### True Belief

The sample was composed of 50 infants (27 boys and 23 girls, *M*_age_ = 16.80 months, range = 15.43–17.99 months). Infants were randomly assigned to one of two conditions: congruent (*n* = 25) or incongruent (*n* = 25). Eight additional infants were tested and excluded from the analyses due to fussiness during the task administration.

#### False Belief

The sample comprised 54 infants (26 boys and 28 girls, *M*_age_ = 16.40 months, range = 15.20–17.80 months). Infants were randomly assigned to one of two conditions: congruent (*n* = 27) or incongruent (*n* = 27). Five additional infants were tested and excluded from the analyses due to fussiness during the task administration.

#### Manipulation Checks

A control condition was used to compare this switch-agent false belief condition with a same-agent false belief condition (i.e., the original paradigm). This sample was previously tested in our laboratory and data from a subsample (*N* = 34) have been published (see Yott and Poulin-Dubois, 2012). This control condition was composed of 48 infants (21 boys and 27 girls, *M*_age_ = 18.75 months, range = 17.16–20.15 months). Infants were randomly assigned to the congruent (*n* = 23) or incongruent (*n* = 25) groups.

To verify how older children and adults process the task, 40 preschoolers (26 males and 14 females, *M*_age_ = 4.42 years, range = 4.00–4.92 years) were recruited. Thirty adults (8 males and 22 females, *M*_age_ = 22.88 years, range = 18.83–32.99 years) were also recruited on a university campus in a large Canadian city. Participants were students enrolled in Psychology (13), Natural Sciences (11), Business/Finance (4), and Exercise Science (2).

## Procedure and Materials

This study was carried out in accordance with the recommendations of the American Psychological Association ethical guidelines. The protocol was approved by the Concordia University Human Research Ethics committee. All parents of the infant and preschool participants gave written informed consent in accordance with the Declaration of Helsinki. Adults also gave written informed consent before participating.

Before the testing period, infants were familiarized to the testing environment. The caregiver gave written informed consent and completed a short demographic questionnaire. At the end of the session, infants received a certificate of merit for their participation and a small gift. Infants’ caregivers were given $20 as compensation for their participation.

The task was administered on a stage-like apparatus (107 × 97 × 104 cm). This apparatus had a back wall (107 × 97 cm) that was separated in four small doors (the right top and bottom doors: 56.5 × 43.5 cm and the left top and bottom doors: 55 × 43.5 cm). As in Onishi and Baillargeon’s (2005) design, these doors permitted the agents to be out of sight of the infants when closed. A yellow box and a green box (14 × 14 × 14 cm each) were placed 37 cm apart at each end of the stage. The boxes had a 14 × 14 cm opening on the side, covered with fabric. The boxes were placed such that the openings face each other. An orange cup (4.5 × 9 × 3 cm) covered in stickers with a magnet inside was used as the toy being manipulated by the agents. Another magnet was placed underneath the stage, such that it could slide across the stage. A Panasonic camera was positioned to focus on the infant’s face, which is displayed on an LCD monitor. An Apple G5 computer was used to live-code infants’ looking behavior using the Habit 2000 program (University of Texas). Infants were seated on a highchair 110 cm from the stage and their caregiver sits behind the infant. If infants refused to sit in the highchair, they sat on their caregiver’s lap (true belief: *n* = 9, false belief: *n* = 13). In these cases, the caregivers wore a sleep mask over their eyes to avoid biasing the infants’ looking behavior.

### True Belief

As in the original version of this task (Onishi and Baillargeon, 2005), infants viewed three familiarization trials, one belief-induction trial, and one test trial. An additional trial was shown before the familiarization trials to introduce the two different agents (E1 and E2) to the infants (i.e., an exposure trial). One agent was dressed in white, wore a white visor and glasses, and had long hair; the other agent was dressed in black, wore a black visor, had no glasses, and had her hair tied up. The color of the agents’ clothing as well as the role the agent played (E1 vs. E2) was counterbalanced, creating four pairings. In this exposure trial, both agents smiled and waved to the infant until the infant looked away for 2 consecutive seconds after looking at the scene for a minimum of 2 cumulative seconds. Infants could look up to 30 s in total. Given that the end of the trial was completely determined on the infant’s response, it is henceforth referred to as “infant-directed.” Between each trial, an attention-getting sound accompanied the rising and lowering of the screen. Infants’ looking was measured during the infant-directed pause that followed each trial, including the exposure pre-trial. These trials ended when the infants (1) looked away from the scene for 2 consecutive seconds after looking at it for a minimum of 2 cumulative seconds, or (2) looked at the scene for 30 cumulative seconds.

**Figure 1:**
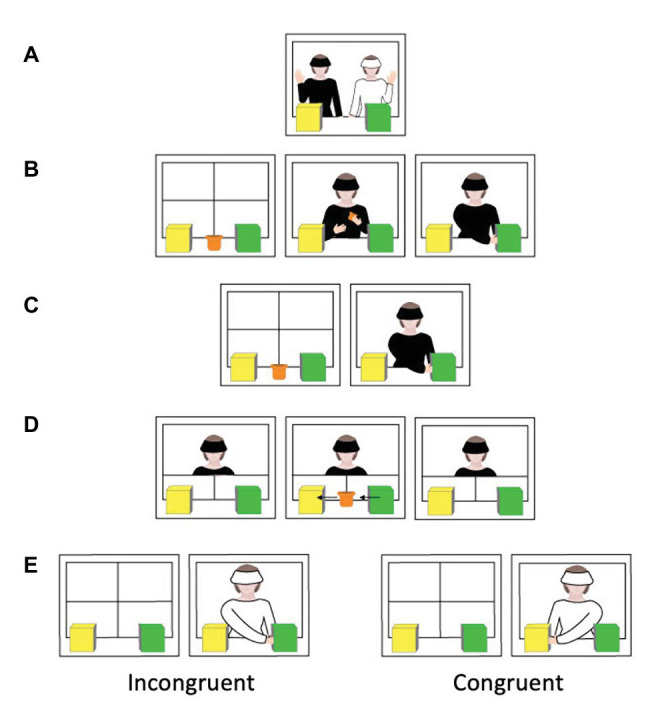
Procedure for the true belief theory of mind (ToM) task. **(A)** Exposure trial, **(B)** first familiarization trial, **(C)** second and third familiarization trials, **(D)** belief-induction trial (true belief), and **(E)** test trial.

The first familiarization trial (12 s) began with the screen rising to reveal E1 sitting (at eye level with the infants) behind two boxes (a yellow box and a green box) and a small cup placed on the table in between the two boxes (see Figure 1). E1 raised her head for a brief moment (approximately 2 s) to ensure that the infants recognized her. E1 then grabbed the toy cup in front of her and gently played with it for 8 s by passing it from hand to hand. After this, E1 placed the toy in the green box and remained in this position until the end of the infant-directed pause. In the second and third familiarization trials (6 s each), after the screen was raised E1 reached and placed her hand inside the green box (i.e., where the cup was hidden) and remained in this position until the end of the infant-directed pause. The goal of these familiarization trials was to show that E1’s goal was to obtain the cup in the green box. During these trials, E2 was raising and lowering the screen following the attention-getting sound.

In the belief-induction trial (8 s), the two bottom doors behind the two boxes were closed such that E1 was now standing behind these doors watching the toy cup move from the green box to the yellow box. The toy cup changed location without the involvement of E1, who observed the change of location in this scene while E2 moved the toy cup using the magnet from under the stage. Once the toy cup was inside the yellow box, the infant-directed pause began, during which E1 kept her gaze on the yellow box (i.e., E1 had a *true* belief that the cup was located in the yellow box). Once this infant-directed pause ended and the curtain was lowered, E1 and E2 switched position, such that E1 was now raising/lowering the curtain and E2 was the agent in the scene. When the test trial (6 s) began, the curtain was raised to reveal E2 sitting behind the two boxes. E2 raised her head for a brief moment to ensure the infants noted the change of agent. Infants in the congruent group saw E2 reach in the yellow box (congruent with E1’s belief) and infants in the incongruent group saw E2 reach in the green box (incongruent with E1’s belief). E2 paused with her hand inside the box until the end of the infant-directed pause. The third experimenter live-coded the infant’s looking time at the scene to transition to the next trial after the infant-directed pauses. Infants’ total looking time (in seconds) at the scene during the infant-directed test pauses was recorded. The waving pre-trial and agent-switch in the test trial excluded, this paradigm was an exact replication of Onishi and Baillargeon’s (2005) VOE task, which was approved by the original author (Baillargeon, personal communication, October 9, 2017).

### False Belief

**Figure 2:**
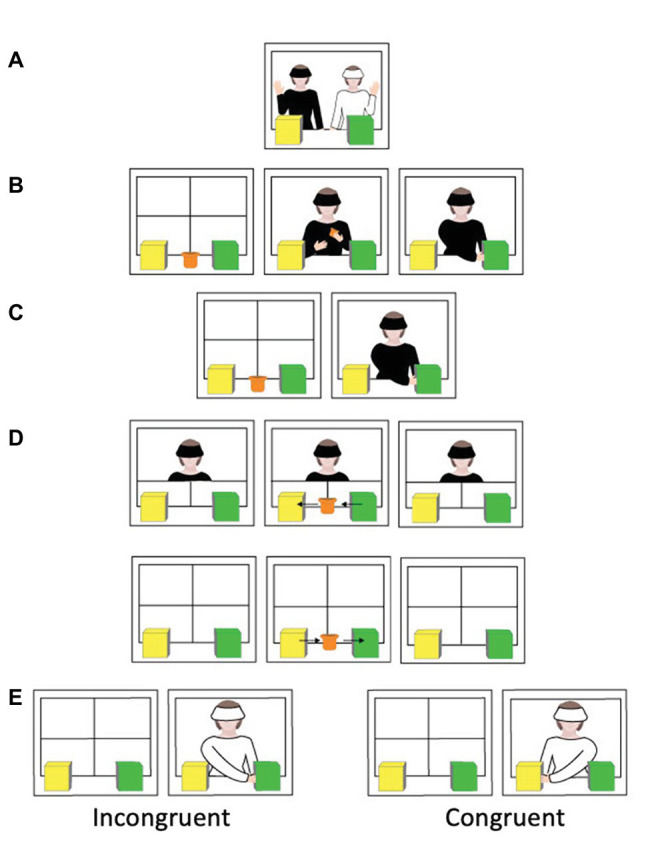
Procedure for the false belief ToM task. **(A)** Exposure trial, **(B)** first familiarization trial, **(C)** second and third familiarization trials, **(D)** belief-induction trial (false belief), and **(E)** test trial.

Infants in the false belief condition saw the same waving pre-trial and three familiarization trials as in the true belief condition. During the belief-induction trial (24 s), infants also saw E1 watch as the toy cup moved to the yellow box (see Figure 2). However, once the toy disappeared inside the yellow box, E1 closed the two upper white doors, thus disappearing from the scene. Following this, the toy cup moved back to the green box (i.e., E1 had a *false* belief that the toy cup was in the yellow box when it was actually located in the green box). The infant-directed pause started once the cup enters the green box. Again, once the screen was lowered at the end of the belief-induction trial, E1 and E2 switched positions. When the test trial (6 s) began, the curtain was raised to reveal E2 positioned behind the two boxes. As in the true belief condition, E2 raised her head for a brief moment then reached either in the yellow box (congruent condition) or in the green box (incongruent condition). Again, infants’ total looking time (in seconds) at the scene during the infant-directed test pauses was recorded by a third experimenter.

### Preschoolers and Adults

The children completed the task at the end of a testing session including other tasks, and received a small gift and a certificate of merit. The parent received $20 to cover travel expenses. Before viewing the pre-recorded video, adult participants completed a demographic questionnaire and were entered in a draw with the possibility of winning a $20 prize. The video consisted of the exposure trial, three familiarization trials, and the induction trial of the false belief switch-agent condition. The video was interrupted after the curtain was raised at the onset of the test trial, showing the naïve agent sitting behind the two boxes. Adults and children were asked the following question “Do you think the actor will search in the yellow box or in the green box?”

### Coding and Reliability

For the VOE task, Habit 2000 was used to live-code the infants’ looking time during the infant-directed pauses. To obtain a more precise measurement, infants’ looking time was recoded offline using INTERACT 8.0 (Mangold) by the primary experimenter. To assess reliability, a second coder who was blind to the hypothesis of the study coded 25% of the video recordings. Cohen’s kappa reliability was 0.85 for the false belief videos and 0.82 for the true belief videos.

## Results

Using z-scores with cut-offs of ±3.0, one participant’s response in the test trial of the congruent condition in the true belief condition and one in the test trial of the congruent condition in the false belief condition were identified as an outlier. These scores were replaced with the next highest value within 3 SDs of the congruent condition mean. Following this modification, the distribution of infants’ looking time at the screen during all trials was normally distributed. Analyses conducted with the samples that included these outlier scores yielded the same results.

### True Belief Condition

First, analyses were conducted to make sure that the infants looked at both agents during the waving pre-trial. On average, infants in the incongruent group looked at the scene during this trial for 20.18 s (*SD* = 9.09) whereas infants in the congruent group looked for 17.46 s (*SD* = 9.80). A 2 (side) × 4 (pairing) ANOVA was conducted to determine if the infants developed an agent/color preference during the waving pre-trial – the side variable refers to infants’ looking time to each side of the stage during this trial given that the position of the two agents was counterbalanced across infants. No main effect of side [*F*(1, 46) = 0.001, *p* = 0.98, *η* ^2^ < 0.001] or pairing [*F*(3, 46) = 1.19, *p* = 0.33, *η* ^2^ = 0.07] nor an interaction [*F*(3, 46) = 0.32, *p* = 0.81, *η* ^2^ = 0.02] were found, indicating that infants looked equally to both agents across all four pairings. In other words, no agent, color, or side preference was found. Next, a 3 (familiarization trials) × 2 (group) ANOVA was used to analyze whether infants in the two groups differed in their pattern of looking during the familiarization trials. A significant main effect of trial was found [*F*(2, 96) = 47.71, *p* < 0.001, *η* ^2^ = 0.50]. No main effect of group [*F*(1, 48) = 2.28, *p* = 0.14, *η* ^2^ = 0.05] nor an interaction [*F*(2, 96) = 0.67, *p* = 0.51, *η* ^2^ = 0.01] were observed. Planned comparisons indicated that infants looked longer during the first familiarization trial (*M* = 15.30 s, *SD* = 7.53 s) than during the second (*M* = 8.81 s, *SD* = 6.25 s; mean difference = 6.49, *p* < 0.001, *d* = 0.94) and third familiarization trials (*M* = 5.92 s, *SD* = 3.71 s; mean difference = 9.38, *p* < 0.001, *d* = 1.58). Further, infants looked longer during the second familiarization trial than during the third familiarization trial (mean difference = 2.89, *p* = 0.01, *d* = 0.56). On average, infants in the incongruent group looked at the scene for 10.97 s (*SD* = 5.35 s) during the familiarization trials and those in the congruent group looked for 9.06 s (*SD* = 3.37 s).

### False Belief Condition

On average, infants in the incongruent group looked at the scene during the waving pre-trial for 19.33 s (*SD* = 10.50) while infants in the congruent group looked for 17.87 s (*SD* = 10.13). Once more, a 2 (side) × 4 (pairing) ANOVA was conducted to determine if the infants developed an agent/color preference during the waving pre-trial. No main effect of side [*F*(1, 50) = 0.51, *p* = 0.48, *η* ^2^ = 0.01] or pairing [*F*(3, 50) = 0.42, *p* = 0.74, *η* ^2^ = 0.02] nor an interaction [*F*(3, 50) = 1.26, *p* = 0.30, *η* ^2^ = 0.07] were found. This indicated that there was no agent, color, or side preference during the waving pre-trial. A 3 (familiarization trials) × 2 (group) ANOVA was conducted to determine if infants’ looking during the familiarization trials differed across the two groups. As in the true belief condition, a significant main effect of trial was found [*F*(2, 104) = 57.40, *p* < 0.001, *η* ^2^ = 0.53]. No main effect of group [*F*(1, 52) = 0.17, *p* = 0.69, *η* ^2^ = 0.003] nor an interaction [*F*(2, 104) = 1.48, *p* = 0.23, *η* ^2^ = 0.03] were found. Planned comparisons indicated that infants looked longer during the first familiarization trial (*M* = 17.89 s, *SD* = 7.56 s) than during the second (*M* = 8.00 s, *SD* = 4.01 s; mean difference = 9.89, *p* < 0.001, *d* = 1.63) and third familiarization trials (*M* = 7.88 s, *SD* = 6.67 s; mean difference = 10.01, *p* < 0.001, *d* = 1.40). There was no difference between infants’ looking in the second and third familiarization trials (mean difference = 0.12, *p* = 1.0, *d* = 0.02). On average, infants in the incongruent group looked at the scene for 11.51 s (*SD* = 5.14 s) during the familiarization trials and those in the congruent group looked for 11.06 s (*SD* = 3.41 s).

### Main Analyses

**Figure 3:**
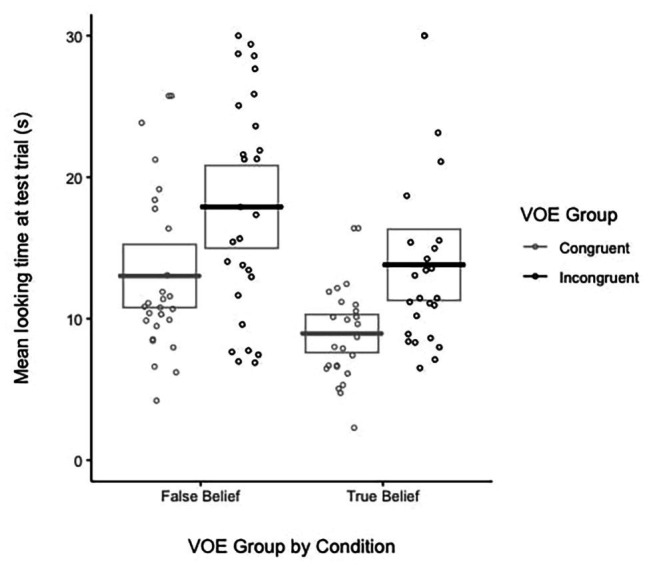
Pirate plots of mean looking time at the test trial (in seconds) in the false and true belief conditions. Boxes represent CIs of the mean, colored lines represent mean looking time at the test trial for each group, and colored points represent individual participants’ looking time at the test trial.

First, a 2 (condition) × 2 (group) ANOVA was conducted and revealed a main effect of condition [*F*(1, 100) = 11.58, *p* = 0.001, *η* ^2^ = 0.10] and a main effect of group [*F*(1, 100) = 16.49, *p* < 0.001, *η* ^2^ = 0.14]. *Post hoc* analyses revealed that infants looked longer in the false belief condition (*M* = 15.47 s, *SD* = 7.26 s) than in the true belief condition [*M* = 11.38 s, *SD* = 5.65 s, *t*(102) = 3.18, *p* = 0.002, *d* = 0.63]. Further, across conditions, infants in the incongruent group looked longer (*M* = 15.94 s, *SD* = 7.36 s) than those in the congruent group [*M* = 11.07 s, *SD* = 5.26 s, *t*(102) = 3.89, *p* < 0.001, *d* = 0.76]. Given that the hypothesis predicted longer looking time for the incongruent than congruent condition, planned comparisons were conducted. In the true belief condition, infants in the incongruent group (*M* = 13.81 s, *SD* = 6.41 s) looked longer than those in the congruent group (*M* = 8.95 s, *SD* = 3.43 s) during the test trial [*F*(1, 48) = 11.18, *p* = 0.002, *η* ^2^ = 0.19]. In addition, in the false belief condition, infants in the incongruent group (*M* = 17.91 s, *SD* = 7.74 s) looked longer than the congruent group (*M* = 13.02 s, *SD* = 5.92 s) during the test trial [*F*(1, 52) = 6.78, *p* = 0.01, *η* ^2^ = 0.12; see Figure 3].

### Manipulation Checks

A 2 (Agent Condition: Same‐ vs. Switch-Agent) × 2 (Group: Congruent vs. Incongruent) ANOVA revealed a main effect of agent condition [*F*(1, 98) = 12.47, *p* = 0.001, *η* ^2^ = 0.12], where infants in the switch-agent condition (*M* = 15.46, *SD* = 7.26) looked longer than the infants in the same-agent condition (*M* = 8.46, *SD* = 4.85). The ANOVA also revealed a main effect of group [*F*(1, 98) = 36.61, *p* < 0.001, *η* ^2^ = 0.27], where infants in the incongruent group (*M* = 14.19, *SD* = 7.72) looked longer than the infants in the congruent group (*M* = 10.06, *SD* = 5.84) across both conditions.

Adults predicted that the agent was equally likely to reach for the yellow box (*n* = 16) than for the green box (*n* = 14). These predictions are not different than what would be expected from chance (binomial *p* = 0.86). Similarly, preschoolers also predicted that the agent was equally likely to reach for the yellow box (*n* = 19) or the green box (*n* = 21; binomial *p* = 0.88).

## Discussion

An issue that has been raised in the context of the infant ToM debate is whether infants’ understanding of beliefs is based on the same mechanisms as those reported for older children and adults, as posited by the mentalistic view. The goal of the present study was to attempt to answer this question using a switch-agent paradigm with the classic VOE task. We aimed to replicate Onishi and Baillargeon’s (2005) methodology with two important modifications: two agents were introduced during a pre-trial and an ignorant agent replaced the knowledgeable agent at the test trial – akin to the paradigm used by Buresh and Woodward (2007). To replicate the original VOE paradigm as closely as possible to limit potential confounds, we designed the study to include an infant-directed exposure trial at the beginning of the VOE paradigm so that the only modification to the VOE paradigm was the ignorant actress at test. We reasoned that if infants have a mature understanding of beliefs, then they should understand that an ignorant agent does not hold the same beliefs as the knowledgeable agent. In other words, infants’ looking patterns should reveal equal looking time for both the congruent and incongruent groups (i.e., no expectations violated). Alternately, we would expect the incongruent group to look longer if the naïve agent is believed to possess the same goals and beliefs as the knowledgeable agent (i.e., shared mental states). Results in both the true and false belief conditions demonstrated that infants generalized the knowledgeable agent’s beliefs to the ignorant agent, who was only present during the test trial. Specifically, infants looked longer, indicating surprise, when the second agent searched for the toy cup in the location that was inconsistent with the first agent’s goals and beliefs. This longer looking observed in the incongruent group indicates that infants’ expectations of the ignorant agent’s actions were violated as they expected her to have a belief based on the knowledgeable agent’s previous behaviors. This finding replicates the looking pattern found by Onishi and Baillargeon (2005), who used only one agent.

The fact that infants experience difficulties in binding beliefs to appropriate agents and in tracking correctly which agent formed a belief challenges the mentalist view of ToM understanding in infancy. If infants possess an understanding of beliefs equivalent to that observed in adults and older children (i.e., mature), they should conclude that the novel agent possesses no belief (true or false) about the object’s location. This is what adults and preschoolers expected when we asked them to predict where the naïve agent would search for the object in the false belief condition. They correctly assumed that without some previous access to objects or events, beliefs cannot be formed unless through interactions with a knowledgeable agent – beliefs are person specific. Of course, the explicit prediction required by the task that they completed was not equivalent to the VOE task and future research should aim to examine how preschoolers and adults behave in the traditional VOE design.

The developmental trajectory of this *full-fledged* understanding of belief remains to be determined. One could speculate that it coincides with the emergence of explicit belief reasoning during the preschool years and future research will be required to address this issue (Wellman et al., 2001). Another possible pathway is that the development of a mature false belief concept requires a sense of self so that the ability to metarepresent one’s own mental states triggers the emergence of the attribution of representations to others (Southgate, 2020). According to this view, without cognitive self-awareness, infants show an altercentric bias in that they orient to others’ focus of attention and encode a belief that does not belong to a specific individual. In other words, the content of the representation (i.e., the belief), and the agent to which the representation is attached, are encoded and updated separately. For example, Kovács et al. (2010, p. 1832) found results that indicated that “the mere presence of social agents is sufficient to automatically trigger online belief computations […]. Once the beliefs have been computed, adults and infants maintain them even in the absence of the agent, presumably for later use in social interactions.” It could also be argued that there is no generalization of beliefs involved in this study, but rather an effect of attributing a belief to the second agent to the perception of an action performed by the first agent, even without generalization of belief. For example, in the experiments of Kovács et al. (2010), the belief of an agent is automatically computed and stored by adults and infants and remains active even in the absence of the agent. Similarly, it could be automatically activated in the presence of any agent during the test phase.

There are other alternative interpretations of the current findings that do not involve processing or even tracking beliefs. In fact, it is also possible that infants do not have any understanding of beliefs at all (see Hutto et al., 2011 for a full taxonomy of minimalist accounts). For example, if behaviors in the VOE paradigm are guided by the simple rule that “people look for an object at the last place they saw it” then the rule could be applied automatically regardless of the identity of the agent at search time (Perner and Ruffman, 2005; Ruffman, 2014). Or, infants could be submentalizing, such that their looking patterns are a result of the violation of the participants’ expectations of superficial associations created in the previous trials (i.e., perceptual novelty of the test trial). In other words, infants could have simply responded to the novelty of the configuration of colors, shapes, and movements (Heyes, 2014a). Although the current design cannot tease apart these alternative interpretations, we can conclude that the present findings require a revision of the rich mentalistic view that assumes that “false-belief understanding emerges early in life and is *robust* and *sophisticated*” (Scott and Baillargeon, 2017, p. 246).

The present results could indicate that infants generalize beliefs broadly across agents—in other words, they might be capable of mentalizing but seem to be attributing mental states too widely (i.e., to any agent). Such interpretation is compatible with recent findings showing that 16-month-olds generalized false belief to a toy crane (Burnside et al., 2019). Although some research has shown that younger infants understand that motivational mental states, such as goals/intentions (Buresh and Woodward, 2007) and preferences (Henderson and Woodward, 2012) are person specific, infants do not seem to apply the same rule in the case of epistemic mental states, such as beliefs. Infants appear to attribute beliefs relatively indiscriminately and automatically. It is possible that infants perceive intentions as person-specific goals or “behavioral tendencies” rather than mental states (Buresh and Woodward, 2007). That is, infants track others’ intentions (mental states) by tracking the physical target to which these intentions are directed. Thus, they have an easier time understanding that one individual’s behavioral tendency is exclusive to that individual because behaviors are observable. Mental states are unobservable and therefore harder to grasp, which is likely why infants have difficulty understanding that *beliefs* are person specific.

In the present study, infants were surprised that the ignorant agent searched in one location (incongruent) over another (congruent); they were not, however, surprised that the ignorant agent was searching at all. Thus, it appears that infants also generalized the knowledgeable agent’s *goals* to the ignorant agent, challenging that infants understand that goals (i.e., motivational states) are person specific. Further, there are mixed reviews regarding infants understanding of the binding properties of preferences (i.e., Moore, 1999; Kampis et al., 2013), indicating that this phenomenon might not be robust before the first year of life. There are mixed findings regarding infants’ understanding of the subjectivity of desires. In one study, infants generalized desires across individuals at 18 months of age unless ostensive communicative cues indicated shared knowledge (Egyed et al., 2013). In another study, the famous broccoli experiment designed by Repacholi and Gopnik (1997) was adapted with the switch paradigm with the same person showing a preference and asking for food or a different requester (Poulin-Dubois and McKoy, 1999). Infants took much longer to offer an object to a naïve requester and tended to give the food that they preferred (crackers) instead of the food preferred by the agent (broccoli). Because belief understanding is known to develop later, it is possible that 16-month-olds’ understanding of this concept is still rudimentary and that the person-specific nature of beliefs takes more time to develop than motivational states. The present findings conflict with the prediction that the ability to bind mental states to specific individuals should emerge after the first year of life, around 13–14 months (Kampis et al., 2013). Future research will be needed to identify the developmental trajectory of the critical ability to encode beliefs as person specific.

Both true and false belief conditions were administered in the present study. The true belief condition permitted an assessment of the seeing = knowing hypothesis recently brought forward by Tomasello (2018) to interpret infants’ behaviors in the VOE paradigm. According to this leaner mentalist view, infants can pass implicit false belief tasks with simple knowledge inference abilities, that is, what an agent sees and does not see. Tomasello (2018) argues that the concept of beliefs is not yet fully formed in infancy, but rather emerges when explicit ToM tasks are succeeded (see Wellman et al., 2001; Wellman and Liu, 2004). Therefore, implicit false belief tasks, such as the one used in the present study, tap into a more rudimentary ToM ability (i.e., knowledge inference). Thus, if one agent sees the cup go to a location, he or she holds knowledge about the toy cup’s location. Infants look longer in the VOE task because their expectations of the agent’s knowledge state are violated. The second agent in the switch agent paradigm is said to be ignorant because she never sees the location of the cup and therefore should not have any knowledge about the location of this toy cup. Therefore, if seeing = knowing theory of Tomasello (2018) is correct, then infants should not have any expectations about the second, naïve agent’s knowledge state. Results from the present study do not support this interpretation.

It is possible that infants believed that they were in a situation of natural pedagogy so that all information they were shown is generalizable to all observers. By the display of ostensive cues (looking at and waving at the infant) during the exposure phase, it could be argued that infants developed the “expectation that the content of the demonstration represents shared cultural knowledge and is generalizable along some relevant dimension to other objects, other occasions or other individuals” (Csibra and Gergely, 2011, p. 1150). Thus, they might expect that all experimenters have the same knowledge about the location of the object. As noted by Apperly and Butterfill (2009), it has been found that “[Infants] do not expect people to acquire beliefs about an object merely by virtue of standing on it, and they do not take close proximity to an object to be a necessary condition for having a belief about it; instead, some kind of purposive interaction with the object appears to be required (O’Neill, 1996; Dunham et al., 2000; Moll and Tomasello, 2006, 2007, p. 957).” Therefore, it is unlikely that the present findings can solely be explained by natural pedagogy. Alternatively, infants might have inferred that the knowledgeable agent communicated to the naïve agent the location of the object. Given that only one adult and no preschooler mentioned communication of the information from the knowledgeable agent to the naïve agent, it is unlikely that infants could have made such inference. A third, simple alternative interpretation is that infants did not detect the change of experimenter from familiarization to test despite the cues manipulated to maximize their distinct physical appearance and the fact that they appeared side by side during the exposure trial. In the midst of a complex social task, they might have only done a cursory check on the agent, assume it is the same given that categorical descriptors are the same, and thus show a form of change blindness. We believe that the direct comparison with a same agent experiment rules out a lack of agent discrimination as an explanation. The fact that infants looked longer during the test trials than those tested in the traditional Same-Agent design indicates that they detected the new agent. Although we are confident from this manipulation check that infants could tell the experimenters apart, future studies might add additional cues (e.g., gender) or make exposure time to the two agents in the initial phase infant-controlled to ensure that the present results are not caused by an artifact.

Taken together, the results of the present study could be interpreted in numerous ways. Importantly, they indicate that if belief processing is present in infancy, it is not as sophisticated as previously believed. Rather, it appears that infants are using an automatic, inflexible cognitive system such that they attribute beliefs implicitly (if they do) broadly to all agents as well as across agents. Nevertheless, the present findings could also be in line with Fenici and Zawidzki’s (2016) interpretation of infants’ responses on implicit false belief tasks which is an elaboration of Butterfill and Apperly’s (2013) minimalistic theory. Specifically, they argue that the infants do not recognize “enduring mental states,” which would be bound to an individual. Instead, they suggest that infants track relational properties of “bouts” of behaviors, which lead to the attribution of goals, which are “non-enduring” to individual agents. In other words, they argue that once a goal is detected by infants, they will behave in accordance to this goal indiscriminately of whom (or what; Burnside et al., 2019) the agent is. Infants are likely observing events in an object-centered manner, such that behaviors about said objects are generalizable to any agent (Brincker, 2014; but see Buresh and Woodward 2007 for conflicting findings). This is a perspective that is situated at the center of the ToM debate spectrum (i.e., middle-of-the-road theory), with submentalizing and the minimalist view at one end and the rich, mentalistic view at the other end. Specifically, as infants develop joint attention, they are gradually able to use person-centered ways to process events, which facilitates perspective-taking such that as children build other skill sets (e.g., language and executive functioning) they are able to *reason* about other individual’s mental states (i.e., in the preschool years; Fenici, 2013; Tomasello, 2018). In the meantime, infants use a more rudimentary, automatic, and broadly applicable belief-tracking ability, which is likely the ability captured by implicit ToM tasks.

In sum, the present study provides additional evidence that the rich, mentalistic view of ToM understanding in infancy should be toned down. Instead, it appears that infants are, in fact, attributing mental states to agents, but too broadly for this ability to be considered as “sophisticated” as in older children and adults. Such broad attribution of mental states is likely adaptive for younger infants, but as they develop, they gradually form more sophisticated understanding of mental states, starting with goals and preferences, and eventually beliefs as children enter the preschool years.

## Data Availability Statement

The raw data supporting the conclusions of this article will be made available by the authors, without undue reservation.

## Ethics Statement

The studies involving human participants were reviewed and approved by Concordia University Human Research Ethics Committee. Written informed consent to participate in this study was provided by the participants' legal guardian/next of kin.

## Author Contributions

All three authors conceptualized the study. CN and KB tested the participants, coded, and analyzed the data. KB wrote a first draft of the manuscript that was edited by CN and DP-D. All authors contributed to the article and approved the submitted version.

### Conflict of Interest

The authors declare that the research was conducted in the absence of any commercial or financial relationships that could be construed as a potential conflict of interest.

## References

[ref1] Apperly I. A. Butterfill S. A. (2009). Do humans have two systems to track beliefs and belief-like states? Psychol. Rev. 116, 953–970. 10.1037/a0016923, PMID: 19839692

[ref3] Baillargeon R. Buttelmann D. Southgate V. (2018). Invited commentary: interpreting failed replications of early false-belief findings: methodological and theoretical considerations. Cogn. Dev. 46, 112–124. 10.1016/j.cogdev.2018.06.001

[ref4] Baillargeon R. Scott R. M. Bian L. (2016). Psychological reasoning in infancy. Annu. Rev. Psychol. 67, 159–186. 10.1146/annurev-psych-010213-115033, PMID: 26393869

[ref5] Baillargeon R. Scott R. M. He Z. (2010). False-belief understanding in infants. Trends Cogn. Sci. 14, 110–118. 10.1016/j.tics.2009.12.006, PMID: 20106714 PMC2930901

[ref6] Baron-Cohen S. Leslie A. M. Frith U. (1985). Does the autistic child have a “theory of mind”? Cognition 21, 37–46. 10.1016/0010-0277(85)90022-8, PMID: 2934210

[ref7] Brincker M. (2014). Navigating beyond “here & now” affordances—on sensorimotor maturation and “false belief” performance. Front. Psychol. 5:1433. 10.3389/fpsyg.2014.01433, PMID: 25566118 PMC4266020

[ref8] Buresh J. S. Woodward A. (2007). Infants track action goals within and across agents. Cognition 104, 286–314. 10.1016/j.cognition.2006.07.001 16930577

[ref9] Burnside K. Severdija V. Poulin-Dubois D. (2019). Infants attribute false beliefs to a toy crane. Dev. Sci. 23:e12887. 10.1111/desc.12887 31309631

[ref10] Butterfill S. A. Apperly I. A. (2013). How to construct a minimal theory of mind. Mind Lang. 28, 606–637. 10.1111/mila.12036

[ref12] Carey S. (2000). The origin of concepts. J. Cogn. Dev. 1, 37–41. 10.1207/S15327647JCD0101N_3

[ref11] Carruthers P. (2013). Mindreading in infancy. Mind Lang. 28, 141–172. 10.1111/mila.12014

[ref13] Clements W. A. Perner J. (1994). Implicit understanding of belief. Cogn. Dev. 9, 377–395. 10.1016/0885-2014(94)90012-4

[ref16] Csibra G. Gergely G. (2011). Natural pedagogy as evolutionary adaptation. Philos. Trans. R. Soc. B 366, 1149–1157. 10.1098/rstb.2010.0319, PMID: 21357237 PMC3049090

[ref14] Dunham P. J. Dunham F. O’Keefe C. (2000). Two-year-old’s sensitivity to a parent’s knowledge state: mind reading or contextual cues? Br. J. Dev. Psychol. 18, 519–532. 10.1348/026151000165832

[ref18] Egyed K. Király I. Gergely G. (2013). Communicating shared knowledge in infancy. Psychol. Sci. 24, 1348–1353. 10.1177/0956797612471952, PMID: 23719664

[ref19] Fenici M. (2013). Social cognitive abilities in infancy: is mindreading the best explanation? Philos. Psychol. 28, 387–411. 10.1080/09515089.2013.865096

[ref20] Fenici M. (2015). A simple explanation of apparent early mindreading: infants’ sensitivity to goals and gaze direction. Phenomenol. Cogn. Sci. 14, 497–515. 10.1007/s11097-014-9345-3

[ref21] Fenici M. (2016). “Succeeding in the false belief test. Why does experience matter?” in New developments in logic and philosophy of science. eds. Felline L. Ledda A. Paoli F. Rossanese E. (UK: College Publications), 77–86.

[ref22] Fenici M. Garofoli D. (2020). An associationist bias explains different processing demands for toddlers in different traditional false-belief tasks. Hum. Dev. 64, 4–6. 10.1159/000505208

[ref23] Fenici M. Zawidzki T. (2016). Action understanding in infancy: do infant interpreters attribute enduring mental states or track relational properties of transient bouts of behavior? Studia Philosophica Estonica 9, 237–257. 10.12697/spe.2016.9.1.10

[ref24] Gergely G. Nádasdy Z. Csibra G. Bíró S. (1995). Taking the intentional stance at 12 months of age. Cognition 56, 165–193. 10.1016/0010-0277(95)00661-H, PMID: 7554793

[ref25] Henderson A. M. Woodward A. L. (2012). Nine-month-old infants generalize object labels, but not object preferences across individuals. Dev. Sci. 15, 641–652. 10.1111/j.1467-7687.2012.01157.x, PMID: 22925512 PMC3430974

[ref26] Heyes C. (2014a). False belief in infancy: a fresh look. Dev. Sci. 17, 647–659. 10.1111/desc.12148 24666559

[ref27] Heyes C. (2014b). Submentalizing: I am not really reading your mind. Perspect. Psychol. Sci. 9, 131–143. 10.1177/1745691613518076 26173251

[ref28] Hutto D. D. Herschbach M. Southgate V. (2011). Editorial: social cognition: mindreading and alternatives. Rev. Philos. Psychol. 2, 375–395. 10.1007/s13164-011-0073-0 PMC3339022 22593773

[ref29] Kampis D. Somogyi E. Itakura S. Király I. (2013). Do infants bind mental states to agents? Cognition 129, 232–240. 10.1016/j.cognition.2013.07.004, PMID: 23942349

[ref31] Kovács A. M. Téglás E. Endress A. D. (2010). The social sense: susceptibility to others’ beliefs in human infants and adults. Science 330, 1830–1834. 10.1126/science.1190792, PMID: 21205671

[ref33] Krupenye C. Call J. (2019). Theory of mind in animals: current and future directions. Wiley Interdiscip. Rev. Cogn. Sci. 10, e1503. 10.1002/wcs.1503, PMID: 31099977

[ref34] Low J. Apperly I. A. Butterfill S. A. Rakoczy H. (2016). Cognitive architecture of belief reasoning in children and adults: a primer on the two-systems account. Child Dev. Perspect. 10, 184–189. 10.1111/cdep.12183

[ref35] Low J. Wang B. (2011). On the long road to mentalism in children’s spontaneous false-belief understanding: are we there yet? Rev. Philos. Psychol. 2, 411–428. 10.1007/s13164-011-0067-y

[ref30] Moll H. Tomasello M. (2006). Level 1 perspective-taking at 24 months of age. Br. J. Dev. Psychol. 24, 603–613. 10.1348/026151005X55370

[ref32] Moll H. Tomasello M. (2007). How 14- and 18-month-olds know what others have experienced. Dev. Psychol. 43, 309–317. 10.1037/0012-1649.43.2.309 17352541

[ref36] Moore C. (1999). “Intentional relations and triadic interactions” in Developing theories of intention: Social understanding and self-control P. eds. Zelazo D. Astington J. W. Olson D. R. (Mahwah, NJ: Erlbaum), 43–61.

[ref42] O’Neill D. K. (1996). Two-year-old children’s sensitivity to a parent’s knowledge state when making requests. Child Dev. 67, 659–677. 10.1111/j.1467-8624.1996.tb01758.x

[ref37] Onishi K. H. Baillargeon R. (2005). Do 15-month-old infants understand false beliefs? Science 308, 255–258. 10.1126/science.1107621, PMID: 15821091 PMC3357322

[ref38] Perner J. Ruffman T. (2005). Infants’ insight into the mind: how deep? Science 308, 214–216. 10.1126/science.1111656, PMID: 15821079

[ref39] Phillips A. T. Wellman H. M. (2005). Infants' understanding of object-directed action. Cognition 98, 137–155. 10.1016/j.cognition.2004.11.005, PMID: 16307956

[ref40] Poulin-Dubois D. McKoy K. (1999). Understanding of the subjectivity of desires in 18-month-olds. Poster presented at the biennial meeting of the Society for Research in child development. Albuquerque, NM.

[ref41] Poulin-Dubois D. Rakoczy H. Burnside K. Crivello C. Dorrenberg S. Edwards K. (2018). Do infants understand false beliefs? We don’t know yet – a commentary on Baillargeon, Buttelmann and Southgate’s commentary. Cogn. Dev. 48, 302–315. 10.1016/j.cogdev.2018.09.005

[ref45] Repacholi B. M. Gopnik A. (1997). Early reasoning about desires: evidence from 14- and 18-month-olds. Dev. Psychol. 33, 12–21. 10.1037/0012-1649.33.1.12 9050386

[ref43] Rubio-Fernández P. Jara-Ettinger J. Gibson E. (2017). Toddlers’ performance in false-belief tasks. Proc. Natl. Acad. Sci. 114:E3750. 10.1073/pnas.1701286114 28416653 PMC5441716

[ref44] Ruffman R. (2014). To belief or not belief: Children’s theory of mind. Dev. Rev. 34, 265–293. 10.1016/j.dr.2014.04.001

[ref49] Scholl B. J. Tremoulet P. D. (2000). Perceptual causality and animacy. Trends Cogn. Sci. 4, 299–309. 10.1016/s1364-6613(00)01506-0 10904254

[ref46] Scott R. M. (2017). The developmental origins of false-belief understanding. Curr. Dir. Psychol. Sci. 26, 68–74. 10.1177/0963721416673174

[ref47] Scott R. M. Baillargeon R. (2014). How fresh a look? A reply to Heyes. Dev. Sci. 17, 660–664. 10.1111/desc.12173, PMID: 24666589

[ref48] Scott R. M. Baillargeon R. (2017). Early false-belief understanding. Trends Cogn. Sci. 21, 237–249. 10.1016/j.tics.2017.01.012, PMID: 28259555

[ref52] Setoh P. Scott R. M. Baillargeon R. (2016). Two-and-a-half-year-olds succeed at a traditional false-belief task with reduced processing demands. Proc. Natl. Acad. Sci. U. S. A. 113, 13360–13365. 10.1073/pnas.1609203113 27821728 PMC5127346

[ref50] Southgate V. (2020). Are infants altercentric? The other and the self in early social cognition. Psychol. Rev. 127, 505–523. 10.1037/rev0000182 31868391

[ref57] Surian L. Franchin L. (2020). On the domain specificity of the mechanisms underpinning spontaneous anticipatory looks in false‐belief tasks. Dev. Sci. e12955. 10.1111/desc.12955 32107820

[ref51] Tauzin T. Gergely G. (2018). Communicative mind-reading in preverbal infants. Sci. Rep. 8:9534. 10.1038/s41598-018-27804-4 29934630 PMC6015048

[ref53] Tomasello M. (2018). How children come to understand false beliefs: a shared intentionality account. PNAS 115, 1–8. 10.1073/pnas.1804761115 PMC6112688 30104372

[ref54] Wellman H. M. (1990). The child’s theory of mind. Cambridge, MA: MIT Press.

[ref55] Wellman H. M. (2014). Making minds: How theory of mind develops. Oxford, UK: Oxford University Press.

[ref56] Wellman H. M. Liu D. (2004). Scaling of theory-of-mind tasks. Child Dev. 75, 523–542. 10.1111/j.1467-8624.2004.00691.x, PMID: 15056204

[ref59] Westra E. Carruthers P. (2017). Pragmatic development explains the theory-of-mind scale. Cognition 158, 165–176. 10.1016/j.cognition.2016.10.021, PMID: 27835787

[ref62] Yott J. Poulin-Dubois D. (2012). Breaking the rules: do infants have a true understanding of false belief? Br. J. Dev. Psychol. 30, 156–171. 10.1111/j.2044-835X.2011.02060.x, PMID: 22429039

